# Why the Public Need a Say in How Patient Data are Used for Covid-19 Responses

**DOI:** 10.23889/ijpds.v5i2.1357

**Published:** 2020-06-23

**Authors:** M Aitken, S Cunningham-Burley, A Darlington, S Elstub, O Escobar, KH Jones, N Sethi, R Thompson

**Affiliations:** 1 Newcastle University, Urban Sciences Building, 1 Science Square, Newcastle upon Tyne, NE4 5TG; 2 Usher Institute, University of Edinburgh, Old Medical School, Teviot Place, Edinburgh, EH8 9AG; 3 Imperial College Health Partners, 30 Euston Square, London, NW1 2FB; 4 Department of Politics, School of Geography, Politics and Sociology, Newcastle University, Newcastle upon Tyne, NE1 7RU; 5 University of Edinburgh, Chrystal Macmillan Building, Edinburgh, EH8 9LD; 6 Swansea University Medical School, Singleton Park, Swansea, SA2 8PP; 7 Centre for Biomedicine, Self and Society, Usher Institute, University of Edinburgh, 23 Buccleuch Place, Edinburgh, EH8 9LN; 8 Data Science Building, Swansea University Medical School, Singleton Park, Swansea, SA2 8PP

 Dear Editors,

The global coronavirus pandemic has clearly demonstrated the great urgency to collect and use patient data effectively to understand, track and manage the spread of Covid-19. The value of patient data in this pandemic is undeniable, however considerations around how – and by whom - such data should be collected, accessed and used, and for what purposes, remain to be fully debated and resolved. Who decides, and how such decisions are made, remain unclear. We argue that, as with all uses of patient data, public engagement and deliberation are essential for good governance and are key to establish and maintain a legitimate social licence for data practices around Covid-19.

Previous data controversies have clearly demonstrated the importance of establishing a social licence for data practices, and that there can be meaningful differences between what is legally permissible and what is socially acceptable [1]. The standard response to such controversies has typically been reemphasising commitments to public engagement [2] in order to (re)build or restore public trust [3]. This overlooks the dynamic nature of public trust, and the importance of ongoing relationships to establish and maintain trust over time.

Commitments to public engagement must go beyond lip service [2] and also need to recognise that not only are there different approaches to public engagement, some are more legitimate and useful than others. We advocate engaging the public, policy makers and users of patient data in collective deliberation to enable mutual learning and informed policy making. This would make the social licence epistemically superior and more legitimate.

We already have a consensus statement on public involvement and engagement (PI&E) relating to data-intensive health research published in IJPDS last year [2]. This statement was co-authored by 31 international researchers, practitioners and patient representatives from the U.K., Ireland, Australia, Canada, Finland and the Netherlands. It sets out eight principles to underpin best practice in this field and to inform the design, implementation and evaluation of PI&E strategies and activities.

The principles put forward in the consensus statement are, that public involvement and engagement with data-intensive health research should:

1. Have institutional buy-in;
2. Have clarity of purpose;
3. Be transparent;
4. Involve two-way communication;
5. Be inclusive and accessible to broad publics;
6. Be ongoing;
7. Be designed to produce impact;
8. Be evaluated.

It is time to reinvigorate these principles so that public engagement is not overlooked in the rapid response to COVID-19.

The key premise of the consensus statement is that the public should not be characterised as a problem to be overcome, but a key part of the solution towards establishing socially beneficial data-intensive health research for all. This resonates in the current context where it is important to avoid caricaturing “the public” or speculating on how “the public” will respond to particular measures or data practices, but rather to engage diverse publics in consequential deliberation to inform and shape policy responses and data practices.

A number of public engagement initiatives relating to Covid-19 have been announced [4] and innovative approaches are being developed to engage the public in these discussions during lockdown and beyond. Innovation in using digital methods is clearly vital if we are to engage diverse publics at this time in the development and governance of new data initiatives. We must also strive to be inclusive of those who are not already online or have little experience of digital communication.

Despite the speed required to deal with the pandemic, it is vital to adhere to these principles not just to do things well in relation to the current crisis, but because current practices, hastily developed, will forge the way for future ways of working. The pandemic brings with it an imperative to realise the value of PI&E in shaping socially acceptable and ethically robust data practices and to raise the profile of PI&E increasing public interest in these activities. This will ensure that the social licence generated will endure because it is based on authoritative and authentic deliberation.

 Yours Sincerely,

Dr Mhairi Aitken, Senior Research Associate, Newcastle University

Prof. Sarah Cunningham-Burley, Professor of Medical and Family Sociology / Dean of Molecular, Genetic and Population Health Sciences, Usher Institute, University of Edinburgh

Amy Darlington, Executive Director, Imperial College Health Partners and Engagement Lead for OneLondon Local Health and Care Record Exemplar

Dr Stephen Elstub, Senior Lecturer in British Politics, Newcastle University

Dr Oliver Escobar, Senior Lecturer in Public Policy, University of Edinburgh

Prof. Kerina Jones, Professor of Population Data Science, Swansea University,

Dr Nayha Sethi, Chancellor's Fellow in Data Driven Innovation, Centre for Biomedicine, Self and Society, University of Edinburgh

Rachel Thompson, Research Associate, Population Data Science, Swansea University 
